# The Effect of Storage Time on the Quality of Low-Sugar Apple Jams with Steviol Glycosides

**DOI:** 10.3390/foods14213678

**Published:** 2025-10-28

**Authors:** Marlena Pielak, Ewa Czarniecka-Skubina

**Affiliations:** Department of Food Gastronomy and Food Hygiene, Institute of Human Nutrition Sciences, Warsaw University of Life Sciences (WULS-SGGW), 159C Nowoursynowska St., 02-776 Warsaw, Poland; marlena_pielak@sggw.edu.pl

**Keywords:** low-sugar apple jams, steviol glycosides, storage, physicochemical parameters, clean label, sugar substitution

## Abstract

This study investigated the effect of storage time on the quality of low-sugar apple jams partially substituted with steviol glycosides (SGs). Apple jams were prepared with 0%, 10%, 20%, 30%, and 40% sugar replacement using highly purified SGs (95.1%). The jams were evaluated immediately after production and after 3 and 6 months of storage at 22 °C in the dark. Physicochemical analyses included dry matter, total soluble solids, vitamin C, total ash, pH, titratable acidity, malic acid, and color parameters (L*, a*, b*). Sensory and microbiological assessments were also carried out. During storage, the dry matter content significantly decreased from 41.4% (control) to 35.6% (40% SGs), while titratable acidity increased from 10.69° to 16.73° (*p* < 0.05), and pH values remained stable (3.15–3.29). Vitamin C content decreased significantly (from 0.56 mg/100 g to 0.19 mg/100 g; 33–66% degradation). The color of jams became lighter with increasing SG substitution (L* increased from 17.19 to 24.73; ΔE up to 9.66) and slightly darkened after storage (ΔL ≈ −1.0). Microbiological analysis confirmed complete safety, with total colony counts < 10 CFU/g and no presence of *Listeria monocytogenes* or coagulase-positive *Staphylococcus*. Sensory evaluation by a trained panel (10 assessors, aged 34–56 years, with similar training in fruit and vegetable preserve evaluation) showed that jams with 10–30% SG substitution maintained desirable apple aroma and sweetness, whereas higher SG levels enhanced metallic odor (0.12–0.95 c.u.) and bitterness (0.2–1.9 c.u.) while slightly reducing apple flavor intensity (*p* < 0.05). Despite these differences, all jams remained acceptable after 6 months of storage. Overall, replacing up to 40% of sucrose with steviol glycosides provided microbiological stability, controlled color changes, and acceptable sensory quality, supporting the production of low-sugar jams in line with clean-label and sustainability trends in modern food technology.

## 1. Introduction

Consuming fruits and vegetables in appropriate amounts is a very important factor influencing the functioning of the human body, including supporting the maintenance of both mental and physical health [1]. The most commonly consumed fruit in Europe is the apple (*Malus domestica*), which is rich in nutrients such as vitamins, polyphenols, and micronutrients that help protect against cardiovascular diseases and other chronic conditions, such as diabetes and cancer [2,3,4,5]. The consumption of apples also has a positive impact on lipid metabolism, body weight reduction, and inflammatory processes in the body [1,6]. Apples are one of the main fruits of the temperate climate zone and play an important role in global production, consumption, and foreign trade of horticultural products. In 2015–2017, the domestic apple harvest averaged 3.1 million tonnes per year, representing about 4% of world production. The apple harvest in Poland in 2018–2022 amounted to 3.1 to 4.3 million tonnes [7,8,9]. In 2021, apple consumption was 11.1 kg per person, and in 2022, it was estimated at over 10 kg per person [10]. In recent years, apple harvests in Poland have been systematically increasing, which has resulted in the need to export or process them (40% to 60%) [7]. Zhu et al. [11] report that global food losses and waste account for about half of the global greenhouse gas emissions from the entire food system [11]. Almost one-third of global food production (about 1.3 billion tonnes) does not reach consumers due to various issues, such as social, cultural, demographic, and environmental factors, e.g., food waste [12]. Fresh apples are a perishable product and should be stored in cool conditions to meet annual demand. However, storing all harvested apples is practically unfeasible, making processing an essential step in preventing their degradation and spoilage. Processing apples allows their flavor to be enjoyed year-round while also helping to reduce food waste, which aligns with the concept of sustainable development and supports Goal 12 of the 2030 Agenda [13], which promotes sustainable consumption and production.

To extend shelf life, preserve the product, and reduce food waste caused by spoilage, various food additives are incorporated into food products. However, many consumers still perceive these additives as potentially risky to health. As a result, they seek products with natural food additives that maintain their original taste and color during processing and storage, without synthetic ingredients [14,15,16]. Consumers are increasingly interested in clean-label products—foods that minimize or eliminate artificial additives, undergo minimal processing, and provide transparent product information [17].

Moreover, the production of steviol glycosides contributes to sustainable food production through economic benefits, environmental sustainability, health advantages, and technological advancements. These factors collectively support a more sustainable and health-conscious food industry [18]. Stevia cultivation and the production of steviol glycosides have a lower environmental impact compared to the production of traditional sugar. Life cycle assessments show that SGs have significantly less effect on global warming potential, land use, and other environmental factors. The substitution of sugar with steviol glycosides supports the idea of sustainable development and aligns with consumer preferences for environmentally friendly products [19].

In the case of processed products such as jams, preserves, and marmalades, sugar (sucrose) is a widely used ingredient that serves both as a sweetener and a preservative. Unfortunately, a significant portion of dietary sugars comes from processed foods. Growing consumer awareness of the negative effects of excessive sugar consumption has increased interest in natural alternatives to sucrose [20,21,22]. A natural alternative may be steviol glycosides, which, in addition to their sweetening properties, also exhibit health-promoting effects, including anti-cariogenic, bactericidal, antifungal properties, and mold-inhibiting properties. They also demonstrate stability in acidic environments and at high temperatures. Therefore, they can be used in fruit preserves to reduce simple sugars, especially in jams with high sugar content, thereby lowering the glycemic index (GI) of the final product [23].

Previous studies [24] have shown that substituting sugar with steviol glycosides in apple preserves containing added pectin and citric acid is possible up to a level of 40% (0.20 g/100 g), without significantly worsening the sensory balance below levels acceptable to consumers, while maintaining a stable physicochemical composition of the preserves. Similar results were obtained in other studies by Pielak and Czarniecka-Skubina [25] for jams with very low sugar content, and therefore lower SG content.

It should be emphasized that SG degradation in various food matrices, including jams, can lead to sensory changes, including reduced sweetness and potential off-flavors [26]. Due to the bitter taste and metallic odor that occur with higher SGs content, it is important to establish the right sugar and SGs ratios and conduct storage studies.

In this study, we hypothesized that increased sugar content, and therefore increased SGs substitution, may have a different impact on the storage quality of apple jams than previously demonstrated. This study is crucial for informed sweetener selection based on its effect on the final physicochemical profile of jams, especially considering the paucity of studies on jams with *Stevia rebaudiana* added after storage. Although SGs are known sweeteners, their long-term impact on the holistic quality—especially sensory changes during storage—of low-sugar fruit jams is less documented [27,28,29].

The aim of the study was to evaluate the effect of storage time on the physicochemical, microbiological, and sensory characteristics of apple jams with reduced sugar content in which steviol glycosides were used. We called them low-sugar jams to distinguish them from very-low-sugar jams.

## 2. Materials and Methods

To ensure a comprehensive assessment of the jams’ quality, a structured research design was developed. The study included systematic measurements of physicochemical properties, microbiological stability, and sensory attributes over time. Experimental studies allowed for an in-depth assessment of the effect of sugar substitution with steviol glycosides on the product’s properties during storage. Samples were analyzed immediately after production (0 months) and then stored for three and six months under controlled conditions (22 °C, dark storage) before further evaluation. The jam was stored in glass jars with a volume of 315 mL. For storage, samples with the highest overall sensory balance scores, along with the control sample, were selected for further evaluation.

### 2.1. Preparation of Apple Jams

In preliminary studies [24], sensory acceptable levels of sugar substitution with SGs in low-sugar jams were established. SGs added in the amount of 0–40% (L0, L10, L20, L30, L40) to sugar allowed jams to be obtained with high sensory evaluation scores; sweet, nectar, and apple taste, as well as harmonizing sensory features, i.e., features characteristic of apple jams. Apple jams with sugar and added steviol glycosides, with the composition presented in Table 1, were prepared under laboratory conditions.

The jam was made from the pulp of Gloster apples (*Malus domestica* ‘Gloster’) from the orchards of WULS-SGGW. Raw material with similar fruit size, healthy, and without mechanical damage was used to prepare the jams. Gloster is a German variety obtained by crossing the Gloskenpfel and Richared Delicious varieties, first imported to Poland in 1979. The flesh of this apple variety is white-green, juicy, firm, fine-grained, aromatic, and wine-like, with a sweet-wine taste [30]. The prepared jams contained 95% of the raw material. The extract level in apples used to prepare jams was 16.3%. The jams contained only sugar added to apple pulp in the control sample, and in the samples with the addition of SGs, it was replaced in the amounts of 10%, 20%, 30%, 40%, assuming that 1 g of sugar corresponds to 0.01 g of steviol glycosides. Jams with reduced sugar content compared to the control variant with sugar only were marked with the abbreviation L. To replace sucrose, steviol glycosides (SGs) were highly purified (95.1%, HPLC) in powdered form with rebaudioside A—40.2% and stevioside—45.4% (Stevia Natura SAS, Riom, France). Rebaudioside A is characterized by 150–350 times greater sweetness than sucrose, shows high sweetening power with a lower level of undesirable aftertaste, and has the most desirable flavor profile; slightly bitter and sour [31,32,33]. Stevioside is 200 to 450 times sweeter than sucrose and is responsible for a bitter and licorice-like aftertaste and odor—enhancing properties. The bitter aftertaste of stevioside is less noticeable when mixed with rebaudioside in equal proportions [34]. The steviol equivalent of the glycosides used was 363 mg/g. Powdered amidated citrus-apple pectin (Agnex, Białystok, Poland) with a degree of esterification of 30–35 (DE) and 15–20% amination (DA) was used for acidification, pH regulation, and acidity stabilization of jams. The pectin solution was prepared in the amount recommended by the producer (3 g per 1 kg of apples). An aqueous citric acid solution (Agnex, Białystok, Poland) was also used, the amount of which was calculated based on the acidity balance (Table 1).

### 2.2. Sensory Analysis

The sensory analysis experts performed the evaluation using a quantitative descriptive analysis (QDA). The evaluation descriptors were determined in preliminary tests by the procedure under PN-ISO 13299:2016 [35]. Quantitative assessment was performed using a 10 cm unstructured graphic scale with word anchors (0–10 c.u.). The evaluation conditions in the sensory laboratory were according to PN-ISO 8589:2007 [36]. The jam samples were all coded. In the first stage of the research, the following parameters were assessed: odor (apple, sweet, nectar, wine, metallic, sharp, ‘other’), taste (sweet, bitter, sour), flavor (apple, nectar, spicy, metallic, bland, astringent, ‘other’), color, and sensory balance in apple jams. After storage, selected apple jams (L0, L10, L20, L30, L40) were evaluated during 3 and 6 months for the following sensory features: color, odor (apple, metallic), taste (sweet, bitter), flavor (apple), consistency, and overall quality. To evaluate the jam samples, the evaluators were provided with definitions of each characteristic [24].

Each session of the laboratory sensory tests was attended by a group of experts consisting of 10 people (out of 20 selected, 12 women and 8 men, aged 34–56 years) with a similar level of training in sensory analysis and significant experience in the evaluation of fruit and vegetable products [37,38]. Panelists received encoded samples, a scorecard, along with definitions of evaluated attributes, and water to clean their taste buds between sessions. Evaluation was repeated three times.

### 2.3. Instrumental Color Measurement

The color measurements of the jams were made using a colorimeter (CM-2300d, Konica-Minolta GmbH, Langenhagen, Germany), with a D65 light source (10° observer angle). The colorimeter was calibrated according to the white calibration plate (Minolta Technical Note 1994, Konica Minolta, Inc., Wrocław, Poland). The color was measured in the L*a*b* system (L*—lightness, a*—the proportion of green or red, b*—the proportion of blue or yellow). The measurements were performed in three repetitions and calculated as follows:

- C*—chroma (color intensity), according to the formula: C* = √[(a*)^2^ + (b*)^2^],
- (Δ)—the differences between individual coordinates were determined by subtracting the measurement results between trials: the value of jams and the raw apple pulp value;
- (ΔC)—color saturation value, according to the formula: (ΔC) = √(Δa*)^2^ + (Δb*)^2^);
- (ΔE)—total color difference value according to the formula:

$$∆E^{*}ab=\sqrt{(∆L*{)}^{2\;}+(∆a*{)}^{2\;}+(∆b*{)}^{2\;}}$$

Interpretation of the color difference between samples was as follows [39]:
0 < ΔE* < 1visually non-recognizable by a standard observer1 < ΔE* < 2visually recognizable only by an experienced observer2 < ΔE* < 3.5visually recognizable by an inexperienced observer3.5 < ΔE* < 5every observer can easily see the differenceΔE* > 5an observer recognizes two different colors

### 2.4. Evaluation of Physicochemical Parameters

The following physicochemical tests were performed in low-sugar apple jams with sugar SGs substitution (L0, L10, L20, L30, L40), at the beginning of storage (0), and after 3 and 6 months of storage. Dry matter content expressed as percentage dry weight was assessed by the weight method, in accordance with the PN-A-75101-02:1990 standard [40]. Total ash content was determined in accordance with the standard PN-90/A-75101-08/Az1:2002 [41]. Titratable acidity was determined according to ISO 750:1998, and the results were expressed in mg g^−1^ of malic acid equivalent (MAE) [42]. The pH was assessed by the potentiometric method using a Knick 913 pH meter in accordance with the PN-EN 1132:1999 standard [43]. The content of total soluble solids was determined by the refractometric method according to the PN-A-75101-02:1990 standard [40]. Vitamin C content was assessed using the HPLC–UV/VIS method described in the previous study [24].

### 2.5. Microbiological Quality Evaluation

Microbiological analyses were performed at the beginning of storage, after 3 and 6 months of storage (Total number of colonies at 30 °C [44] using growth medium: Plate Count Agar (PCA) (LabM, Heywood, UK) and incubation at 30 °C for 72 h.

*Listeria monocytogenes* [45,46] was assessed with pre-incubation: half-Fraser and Fraser broth (Oxoid, Waltham, MA, USA) and incubation at 37 °C for 24 h and for 24–48 h, respectively. Growth media: ALOA (Agar Listeria according to Ottaviani and Agosti; LabM, Heywood, UK) and incubation at 37 °C for 24 h. In the case of a positive result on the ALOA, a confirmation result was obtained on the PALCAM agar (LabM, Heywood, UK) with incubation at 37 °C for 24 h.

*Coagulase-positive staphylococci* [47] were assessed in growth medium: Baird–Parker agar medium with 5% Egg Yolk Tellurite (LabM, Heywood, UK) with incubation at 37 °C for 24 h.

Yeast and mold counts [48] were evaluated with growth medium: YGC agar (Sabouraud Dextrose with Chloramphenicol LAB-Agar) with incubation at 25 °C for 5–7 days.

### 2.6. Statistical Analysis

The results were analyzed using the STATISTICA software version.13.1 PL (StatSoft, Kraków, Polska). The results of the physicochemical analysis were presented as mean with standard deviation (SD), whereas the sensory analysis results were presented as standard error (SE). One-way analysis of variance (ANOVA) and Fisher’s least significant difference (LSD) post hoc test for analysis of the results of physicochemical tests and sensory evaluations. Statistical significance was defined at *p* ≤ 0.05.

## 3. Results

### 3.1. Sensory Analysis of Low-Sugar Apple Jams with Steviol Glycosides

Apple-based products with 0–40% substitution (L0, L10, L20, L30, L40) were characterized by a high intensity of sweet, nectar, and apple flavors, as well as a sweet aroma. The substitution of sucrose with SGs influenced an increase in negative attributes, such as astringency and metallic aroma. The most characteristic and easy-to-assess features of apple jams are taste (sweet, bitter), apple flavor, odor (apple, metallic), color, consistency, and overall quality. Therefore, these attributes were used in this jam storage study. These results were presented in the wider context of our previous study as a PCA analysis [24], but now we repeated only the main conclusion to better understand the selection of sensory attributes and changes in low-sugar apple jams with steviol glycosides after storage.

The sensory evaluation revealed that apple jams with steviol glycosides maintained a high level of acceptability among panelists. The samples, regardless of the addition of steviol glycosides, exhibited similar characteristics and were closely comparable to each other (*p* > 0.01). The substitution of sugar with SGs up to 40% had a minimal negative impact on the overall perception of sweetness, as most samples were described as pleasantly sweet and well-balanced. However, an increase in SG concentration correlated with a rise in perceived metallic and bitter notes, which became more pronounced over time. Apple flavor intensity remained strong across all samples, with the highest scores observed in jams with 10–30% SG substitution. Higher substitution levels (above 30%) resulted in slightly altered flavor profiles, with some assessors noting a reduction in the characteristic apple taste. In contrast, jams with lower sugar levels demonstrated a greater prominence of nectar and wine—like flavors, enhancing the overall complexity of the product.

### 3.2. The Effect of Storage on the Sensory Quality of Low-Sugar Apple Jams with Added SGs

The perceived metallic aroma of apple-based products significantly increased with the rising concentration of steviol glycosides (*p* < 0.001) and with the extension of storage time (*p* < 0.05), ranging from 0.12 c.u. to 0.95 c.u. (Table 2). In terms of apple aroma, the low-sugar products were rated highly, with scores ranging from 6.32 c.u. to 8.23 c.u., and storage time did not significantly affect the evaluated samples with SGs substitution of 0%, 10%, and 20%. Statistically significant differences were observed for higher levels of SGs substitution (30% and 40%). The color was rated in the range of 3.70 c.u.–4.63 c.u., with the highest rating observed for the product with 30% steviol glycoside substitution (L30) (Table 2). This variant also had the highest overall quality, and no statistically significant differences in bitterness were found after 3 and 6 months of storage. Bitterness was rated in the range of 0.2 c.u.–1.90 c.u., depending on the substitution level and storage time (Table 2). No statistically significant differences were observed in sweetness perception after storage (*p* > 0.05). For most evaluated samples (with SGs substitution of 10%, 20%, and 30%), no statistically significant differences in apple flavor were detected after the storage period (*p* > 0.05).

The color of apple-based products varied depending on the sugar and steviol glycoside content—the higher the sugar content, the darker the samples appeared. When ΔE was more than 5, observers recognized two different colors between samples with different levels of SGs substitution. The samples were characterized by a yellow hue (positive Δb value). After storage, all samples exhibited darkening (negative ΔL value) and a reduction in yellow color intensity (decrease in Δb value). At the beginning of storage, the samples were greener rather than redder (negative Δa value) compared to the control sample. However, after 3 and 6 months of storage, the color shifted towards a redder shade (positive Δa value). All parameter changes (including ΔE) were minor compared to the jam at the beginning of storage, as the addition of pectin and citric acid contributed to a lighter product color and stabilized color changes during storage (Table 3). The higher the level of SGs substitution from 10% to 40%, the more pronounced the differences between the samples of low-sugar apple jams, as evidenced by the value (ΔE) ***—total color difference value between jams with sugar (control) and various SGs substitution (10%, 20%, 30%, 40%). Jam samples with SGs had higher lightness (L*) than jam samples with only sugar. This is related to the color changes that occur when the sugar is heated and indicates the higher color stability of steviol glycosides under thermal treatment.

### 3.3. The Effect of Storage on the Physicochemical Parameters of Low-Sugar Apple Jams with Added SGs

The total solids content measured by using the refractometric method differed between jam samples with various SGs substitution (0%, 10%, 20%, 30%, 40%) between 45.2% (with 10% SGs), 43.9% (with 20% SGs), 39.0% (with 30% SGs), and 36.0% (with 40% SGs), without significant changes after storage (*p* > 0.05). The storage process of the products for 3 and 6 months caused statistically significant changes in the dry matter content in the case of low-sugar preserves, especially after 6 months of storage. After the storage period, a higher total ash content was observed, indicating minor transformations—the higher the substitution of steviol glycosides (and, therefore, the lower the sucrose content), the greater the total ash content. The pH values remained at a similar level; however, total acidity increased (*p* < 0.05). The vitamin C content in all apple-based products was low. Nevertheless, no strong correlation was found between the amount of vitamin C and the concentration of steviol glycosides (Table 4). The storage process of low-sugar apple preserves for 3 and 6 months contributed to the degradation of vitamin C in all samples, ranging from 33 to 66%.

### 3.4. The Effect of Storage on the Microbiological Quality of Low-Sugar Apple Jams with Added Steviol Glycosides

The microbiological quality evaluation was carried out after initial preparation of apple jams (0—without storage, control sample) and after storage for 3 and 6 months. The results did not show the presence of coagulase-positive staphylococci and *Listeria monocytogenes*. At the beginning and after storage for 3 and 6 months, the content of yeast, mold, and the total number of microorganisms (OLD at 30 °C) was satisfactory, below 10, regardless of the level of sugar substitution with SGs, which is in accordance with food law [49].

### 3.5. Summary

The storage time significantly (*p* < 0.05) affected selected quality parameters of all products (dry matter, total solids content, vitamin C, total Ash, pH, titratable acidity, malic acid, and instrumental color). Despite the stabilization of color changes, an increase in the ΔL parameter and acidity was observed, while microbiological safety was maintained during the 3- and 6-month storage period. The overall sensory balance remained at an acceptable level, with slight differences depending on the degree of sugar substitution. However, a decrease in apple flavor intensity and an increase in metallic odor and bitter taste were noticeable after 3 and 6 months of storage of low-sugar apple jams with SGs (*p* < 0.05).

## 4. Discussion

Apple preserves made from the Gloster variety have a basic chemical composition (dry matter, vitamin C, total acidity, pH, ash) similar to their traditional counterparts. Despite a significant reduction in sugar content by replacing it with steviol glycosides, these products were rated by the assessors as sensory acceptable. As stated in previous studies [24], in low-sugar apples, the substitution of sugar by SGs is possible only up to 40% due to the unacceptable sensory quality, whereas in very-low-sugar apple jams, substituting it with steviol glycosides is possible up to 80% [25].

Low-sugar apple preserves with steviol glycosides stored for 3 and 6 months at 22 °C maintained good microbiological quality throughout the storage period. However, storage time significantly affected the quality of low-sugar apple jams made with steviol glycosides, influencing both physicochemical properties and sensory attributes.

Storage had a moderate effect on sensory characteristics. Over the six-month period, sweetness intensity decreased slightly in all variants, accompanied by a minor increase in sourness and bitterness, particularly in jams with higher SG content. Despite this, most samples retained their desirable attributes, suggesting that steviol glycosides effectively maintain an acceptable sensory profile over time.

An increase in metallic/bitter notes has been noted in apple jams.

The bitter taste may result from the ratio of rebaudioside A and stevioside in the preparation. The most favorable flavor profile is achieved with preparations containing highly purified rebaudioside A (99%). The bitter aftertaste of stevioside is less noticeable when mixed with equal amounts of rebaudioside A [28]. To reduce the bitter taste of stevia, preparations with a high content of rebaudioside A should also be used [50]. The mixture used consisted of rebaudioside A (40.2%) and stevioside (45.4%), which could have influenced the bitter taste, which was even more pronounced after storage. The sweet taste did not change significantly, as in high-acidity products, steviol glycosides can gradually hydrolyze to steviol at elevated temperatures. This does not result in a loss of sweetness, as the resulting steviol also possesses strong sweetening properties. However, there is a lack of studies on long-term storage that would take into account all the changes that occur. Studies indicate that while these jams maintain satisfactory quality for up to six months, certain changes occur over time, particularly in terms of color and bioactive components. Physicochemical analysis showed that storage time had a minor impact on pH levels but led to an increase in titratable acidity.

During low-sugar apple jam with SGs storage, the pH decreases due to increased acidity, which leads to pectin breakdown during storage. This acidification process may enhance microbial suppression in some cases and can also adversely affect the organoleptic properties, for example, flavor, as indicated by other authors [51]. Due to this fact, it is important to monitor the pH and titratable acidity as critical indicators of both microbial stability and sensory quality during storage.

Significant degradation of vitamin C was observed (33–66%). The significant decrease in vitamin C content during 3 months of storage at room temperature can also be attributed to the mutual degradation between anthocyanins and vitamin C. However, apple jams are not a main source of vitamin C in a diet.

The color of the jams also changed over time, with a slight shift towards darker and redder hues. The lightness of apple jam changed from the initial yellow to a reddish color during storage. This was probably related to the formation of brown pigments in the Maillard reaction. Similar results for apricot jam were obtained by Touati et al. [52] for jams only with sugar.

The progressive color degradation in all the jam samples during storage for 3 and 6 months indicates the susceptibility of fruit pigments (anthocyanins) to oxidation and degradation. That is why it is so important to ensure appropriate storage conditions to maintain the quality of the product. Similar results are reported by other authors [53].

Microbiological analysis confirmed that all jam variants remained stable and safe for consumption throughout the storage period. Other authors also report that long-term storage leads to a decrease in total sugar content, free amino acids, and ascorbic acid, indicating a decline in quality [53]. In storage studies, Haroon et al. [54] demonstrated that all sensory characteristics of low-sugar apple jams, including those containing steviol glycosides, deteriorate over time, highlighting the impact of storage duration on product quality.

Ali et al. [55] found that the nutritional profile of jams with added steviol glycosides remains stable for one month at room temperature and can serve as a healthier alternative for individuals with hypertension and diabetes.

In summary, replacing sugar with steviol glycosides in jam production can offer significant health benefits, maintain acceptable sensory properties up to 40% substitution, and ensure good technological properties, making it a viable option for producing healthier, low-calorie jams that align with consumer preferences for natural ingredients.

The use of natural sweeteners such as SG aligns with the clean-label trend, where consumers prefer products with natural ingredients and fewer artificial additives. Additionally, this supports the idea of sustainability and meets consumer demands for environmentally friendly products.

While the sensory quality remains acceptable, further research is needed to refine formulations to mitigate any adverse taste changes and enhance product stability over extended storage periods. Utilizing innovative formulation methods, such as combining SGs with other natural sweeteners, could further increase product appeal and improve overall sensory characteristics, as a viable sugar substitute in low-sugar apple jams, meeting consumer demand for healthier, more natural products.

The evaluation period, during which physicochemical properties such as moisture, ash, pH, total soluble solids, and acidity were monitored, provided preliminary data on the jam’s stability. Although some changes occurred (e.g., decreased moisture and pH, increased TSS and acidity), these are important parameters for determining appropriate shelf life and packaging requirements for commercial distribution. The microbiological evaluation showed that the food was still safe, despite the reduction in sugar, which plays a preservative role in jams, which is crucial for commercial products.

Similar results were found in studies by other authors who demonstrated the effect of storage conditions (temperature, storage time) on the physicochemical properties of apple jam, including the content of dry matter, total sugars, free amino acids, and ascorbic acid, as well as the reduction in their content.

These changes were the result of changes in the individual components of jam and their impact on nutritional stability. Other authors point to similar results. For example, orange jam stored at 25 °C and 35 °C showed a decrease in total sugar content of 12.5% and 15%, respectively, within 30 days, which is related to a decrease in dry matter content due to sugar degradation [56]. In turn, in another publication, focusing on red beetroot jam, it was found that the total content of phenols, flavonoids, and betalains, which are components of dry matter, decreased during storage [57].

## 5. Conclusions

The results of this study include good color stability, particularly its brightness (apple jams tend to darken during storage), reduced sugar content, and maintenance of technological quality after three and six months of storage. The assessed jams containing sugar and substitution levels of SGs (10%, 20%, 30%, and 40%) demonstrate similarity between the evaluated jams and traditional ones, while simultaneously reducing sugar content and achieving a taste preferred by consumers. The good stability of the jams’ basic parameters bodes well for their use in industrial production. All these factors may contribute to higher consumer acceptance of jams with SG compared to existing sugar substitutes, as stevia is a natural product.

Jams with steviol glycoside substitution would be a product with reduced sugar content, aligning with the concept of sustainable development and the clean-label trend, thereby meeting consumer needs.

In summary, replacing sugar with steviol glycosides can provide health benefits, allowing for sugar reduction while maintaining acceptable sensory attributes and good technological properties. Moreover, this approach aligns with the clean-label trend, meeting growing consumer preferences for natural and health-promoting ingredients. The study provides a strong foundation for the commercial production of apple jam with steviol glycosides, highlighting its potential as a healthy product. However, commercialization would require further optimization of storage conditions and extension of shelf life (over 6 months) to ensure widespread consumer acceptance and consistent quality. As these were model laboratory studies in which higher-purity steviol glycosides were used to produce apple jams, this may have reduced the perception of bitter and metallic aftertaste that occurs with lower-quality steviol glycosides, typically used in industrial practice. Currently, there is an increasing interest among consumers in products with reduced sugar content, including jams. New, natural sugar substitutes, such as steviol glycosides, are also being sought, in line with new trends in clean-label food production.

The methods of obtaining steviol glycosides are also changing, which may eliminate their disadvantages, such as bitterness. For this reason, further research should be conducted on the addition of steviol glycosides to the matrix of fruit jams.

## Figures and Tables

**Table 1:** Ingredients for making low-sugar (L) apple jams with steviol glycosides.

Variant Low-Sugar (L) Jam	Sugar (%)	Steviol Glycosides		Pectin (g 100 g^−1^)	Citric Acid (g 100 g^−1^)
		[g 100 g^−1^]	(% *)		
Control sample (L0)	50	0	0	0.3	1.00
L10	45	0.05	10	0.3	0.94
L20	40	0.10	20	0.3	0.89
L30	35	0.15	30	0.3	0.83
L40	30	0.20	40	0.3	0.77

* Percentage of sugar substitution with steviol glycosides.

**Table 2:** Changes in the sensory quality of low-sugar apple jams with steviol glycosides.

SGs (%)	Storage (Month)	Average Value (c.u.) ± SE							
		Taste		Flavor	Odor		Color	Consistency	Overall Quality
		Sweet	Bitter	Apple	Apple	Metallic			
0	0 3 6	8.80 ± 0.40 ^a^ 8.50 ± 0.50 ^a^ 8.40 ± 0.10 ^a^	0.30 ± 0.03 ^a^ 0.20 ± 0.05 ^b^ 0.30 ± 0.02 ^a^	7.30 ± 0.20 ^a^ 7.20 ± 0.20 ^a^ 7.00 ± 0.40 ^a^	6.32 ± 0.08 ^a^ 6.35 ± 0.05 ^a^ 6.40 ± 0.02 ^a^	0.12 ± 0.02 ^a^ 0.15 ± 0.05 ^ab^ 0.20 ± 0.05 ^b^	3.80 ± 0.10 ^a^ 3.90 ± 0.10 ^ab^ 4.20 ± 0.20 ^b^	8.50 ± 0.10 ^a^ 8.00 ± 0.20 ^b^ 7.80 ± 0.10 ^b^	8.50 ± 0.30 ^a^ 8.20 ± 0.20 ^a^ 8.00 ± 0.40 ^a^
10	0 3 6	8.80 ± 0.20 ^a^ 8.60 ± 0.30 ^a^ 8.50 ± 0.10 ^a^	0.30 ± 0.03 ^a^ 0.40 ± 0.04 ^b^ 0.40 ± 0.04 ^b^	7.20 ± 0.20 ^a^ 7.00 ± 0.30 ^a^ 7.00 ± 0.20 ^a^	6.25 ± 0.05 ^a^ 6.20 ± 0.02 ^a^ 6.25 ± 0.05 ^a^	0.66 ± 0.03 ^a^ 0.65 ± 0.03 ^a^ 0.68 ± 0.03 ^a^	3.70 ± 0.10 ^a^ 3.80 ± 0.10 ^a^ 4.00 ± 0.40 ^a^	6.50 ± 0.30 ^a^ 6.30 ± 0.1 ^ab^ 6.00 ± 0.20 ^b^	7.00 ± 0.20 ^a^ 6.70 ± 0.50 ^ab^ 6.50 ± 0.40 ^b^
20	0 3 6	6.86 ± 0.40 ^a^ 6.52 ± 0.30 ^a^ 6.30 ± 0.30 ^a^	0.13 ± 0.02 ^a^ 0.20 ± 0.02 ^a^ 0.20 ± 0.04 ^a^	7.70 ± 0.20 ^a^ 7.20 ± 0.20 ^a^ 7.00 ± 0.30 ^a^	8.23 ± 0.05 ^a^ 8.21 ± 0.02 ^a^ 8.19 ± 0.02 ^a^	0.43 ± 0.03 ^a^ 0.41 ± 0.02 ^a^ 0.40 ± 0.02 ^a^	4.63 ± 0.40 ^a^ 4.50 ± 0.50 ^a^ 4.50 ± 0.40 ^a^	6.60 ± 0.20 ^a^ 6.50 ± 0.30 ^a^ 6.30 ± 0.10 ^a^	7.00 ± 0.20 ^a^ 6.80 ± 0.20 ^ab^ 6.50 ± 0.30 ^b^
30	0 3 6	8.50 ± 0.20 ^a^ 8.60 ± 0.20 ^a^ 9.00 ± 0.50 ^a^	0.20 ± 0.02 ^a^ 0.20 ± 0.04 ^a^ 0.20 ± 0.03 ^a^	7.60 ± 0.10 ^a^ 7.30 ± 0.10 ^b^ 7.20 ± 0.10 ^b^	7.37 ± 0.03 ^a^ 7.30 ± 0.01 ^a^ 7.25 ± 0.05 ^b^	0.58 ± 0.02 ^a^ 0.62 ± 0.02 ^a^ 0.65 ± 0.03 ^b^	4.40 ± 0.40 ^a^ 4.50 ± 0.50 ^a^ 4.50 ± 0.30 ^a^	7.00 ± 0.40 ^a^ 6.80 ± 0.5 ^ab^ 6.50 ± 0.30 ^b^	7.00 ± 0.20 ^a^ 6.80 ± 0.20 ^ab^ 6.50 ± 0.40 ^b^
40	0 3 6	7.90 ± 0.30 ^a^ 7.80 ± 0.20 ^a^ 7.70 ± 0.10 ^a^	1.90 ± 0.04 ^a^ 1.80 ± 0.05 ^b^ 1.8 ± 0.40 ^ba^	6.80 ± 0.30 ^a^ 6.50 ± 0.40 ^ab^ 6.40 ± 0.10 ^b^	6.56 ± 0.04 ^a^ 6.45 ± 0.05 ^b^ 6.40 ± 0.05 ^b^	0.93 ± 0.05 ^ab^ 0.87 ± 0.05 ^a^ 0.95 ± 0.05 b	3.80 ± 0.20 ^a^ 3.90 ± 0.20 ^a^ 4.00 ± 0.10 ^a^	6.50 ± 0.50 ^a^ 6.10 ± 0.1 ^ab^ 5.90 ± 0.30 ^b^	6.80 ± 0.30 ^a^ 6.50 ± 0.50 ^ab^ 6.30 ± 0.10 ^b^

a, b—samples marked with the same letters (in columns differences between storage periods) do not differ statistically significantly (*p* > 0.05); c.u.—conventional unit; SE—standard error.

**Table 3:** Color parameters and differences compared to the control sample (L0) at the beginning of storage of low-sugar (L) apple jams with SGs.

Color (L*a*b*)	Addition of SGs (%) to Low-Sugar (L) Jams				
	0	10	20	30	40
Color at the Beginning of Storage *					
L*	17.19 ± 0.60	22.27 ± 0.18	23.03 ± 0.60	23.94 ± 0.70	24.73 ± 2.29
ΔL	-	5.08	5.84	6.75	7.54
a*	0.23 ± 0.24	−0.39 ± 0.02	−0.42 ± 0.04	−0.47 ± 0.04	−0.49 ± 0.06
Δa	-	−0.62	−0.65	−0.7	−0.72
b*	7.56 ± 0.28	13.22 ± 0.24	13.35 ± 0.42	13.37 ± 0.39	13.55 ± 0.82
Δb	-	5.66	5.79	5.81	5.99
C	7.56	13.22	13.36	13.38	13.56
ΔC	-	5.69	5.83	5.85	6.03
(ΔE)	-	7.63	8.25	8.93	9.66
Color changes after 3 months of storage **					
L*	17.12 ± 0.49	22.11 ± 0.17	22.70 ± 0.41	23.74 ± 0.36	24.13 ± 0.03
ΔL	−0.07	−0.16	−0,33	−0.20	−0.60
a*	0.20 ± 0.07	−0.36 ± 0.09	−0.40 ± 0.05	−0.44 ± 0.01	−0.47 ± 0.02
Δa	−0.03	0.03	0.02	−0.05	0.02
b*	7.24 ± 0.28	13.16 ± 0.13	13.22 ± 0.13	13.27 ± 0.26	13.42 ± 0.26
Δb	−0.32	−0.06	−0.13	−0.10	−0.13
C	7.56	13.16	13.23	13.28	13.43
ΔC	0.03	0.07	0.13	0.11	0.11
(ΔE)	0.03	0.17	0.36	0.23	0.23
(ΔE) ***	-	7.74	8.18	8.89	9.35
Color changes after 6 months of storage **					
L*	17.10 ± 0.15	21.8 ± 0.58	22.30 ± 0.25	23.14 ± 0.7	23.70 ± 0.22
ΔL	−0.09	−0.47	−0.73	−0.8	−1.03
a*	0.18 ± 0.03	−0.35 ± 0.15	−0.40 ± 0.20	−0.42 ± 0.21	−0.46 ± 0.04
Δa	−0.05	0.04	0.00	−0.03	0.03
b*	7.21 ± 0.04	13.12 ± 0.04	13.2 ± 0.04	13.23 ± 0.05	13.3 ± 0.05
Δb	−0.35	−0.10	−0.02	−0.14	−0.14
C	7.56	13.12	13.21	13.24	13.31
ΔC	0.35	0.11	0.02	0.14	0.25
(ΔE)	0.36	0.48	0.73	0.81	1.06
(ΔE) ***	-	7.55	7.94	8.53	8.98

In Table average value ± SD, * color changes compared between different levels of SGs and jam with sugar only (control sample), ** color changes compared to jam with different SGs substitution at the beginning of storage, *** total color difference value between jams with sugar (control) and various SGs substitution (10%, 20%, 30%, and 40%).

**Table 4:** Changes in selected physicochemical parameters in low-sugar apple jams with SGs.

SGs (%)	Time of Storage	Average Value ± SD					
		Dry Matter (%)	Vitamin C (mg/100 g)	Total Ash (%)	pH	Titratable Acidity (°)	Malic Acid (g/100 g)
0	0 3 6	41.4 ± 0.2 ^aA^ 40.6 ± 0.5 ^b^ 40.2 ± 0.7 ^b^	0.56 ± 0.02 ^aA^ 0.37 ± 0.002 ^b^ 0.19 ± 0.01 ^c^	0.161 ± 0.02 ^aA^ 0.129 ± 0.03 ^b^ 0.221 ± 0.138 ^c^	3.29 ± 0.02 ^aA^ 3.22 ± 0.01 ^b^ 3.20 ± 0.01 ^b^	10.69 ± 0.04 ^aA^ 12.70 ± 0.03 ^b^ 16.72 ± 0.02 ^c^	0.71 ± 0.00 ^aA^ 0.85 ± 0.01 ^b^ 1.12 ± 0.02 ^c^
10	0 3 6	40.4 ± 0.1 ^aB^ 40.3 ± 0.3 ^a^ 40.0 ± 0.7 ^a^	0.54 ± 0.02 ^aA^ 0.33 ± 0.01 ^b^ 0.20 ± 0.01 ^c^	0.163 ± 0.01 ^aA^ 0.160 ± 0.01 ^a^ 0.143 ± 0.02 ^b^	3.26 ± 0.01 ^aA^ 3.20 ± 0.01 ^b^ 3.17 ± 0.01 ^c^	12.12 ± 0.04 ^aB^ 14.13 ± 0.12 ^b^ 18.17 ± 0.28 ^c^	0.81 ± 0.00 ^aB^ 0.95 ± 0.01 ^b^ 1.22 ± 0.03 ^c^
20	0 3 6	40.4 ± 0.2 ^aB^ 39.2 ± 0.2 ^a^ 39.6 ± 0.1 ^b^	0.54 ± 0.01 ^aA^ 0.35 ± 0.02 ^b^ 0.23 ± 0.01 ^c^	0.164 ± 0.01 ^aA^ 0.160 ± 0.01 ^a^ 0.155 ± 0.02 ^b^	3.25 ± 0.01 ^aA^ 3.20 ± 0.01 ^b^ 3.15 ± 0.01 ^c^	13.20 ± 0.14 ^aB^ 14.00 ± 0.11 ^b^ 17.27 ± 0.38 ^c^	0.87 ± 0.01 ^aB^ 0.99 ± 0.01 ^b^ 1.12 ± 0.02 ^c^
30	0 3 6	38.6 ± 0.3 ^aC^ 38.4 ± 0.5 ^a^ 40.1 ± 0.1 ^b^	0.53 ± 0.01 ^aA^ 0.33 ± 0.01 ^b^ 0.22 ± 0.02 ^c^	0.167 ± 0.00 ^aA^ 0.174 ± 0.02 ^a^ 0.214 ± 0.01 ^b^	3.21 ± 0.01 ^aB^ 3.17 ± 0.01 ^b^ 3.15 ± 0.01 ^b^	14.98 ± 0.02 ^aC^ 15.77 ± 0.17 ^b^ 17.34 ± 0.48 ^c^	1.00 ± 0.00 ^aC^ 1.06 ± 0.01 ^b^ 1.11 ± 0.08 ^c^
40	0 3 6	34.7 ± 0.3 ^aD^ 35.0 ± 0.2 ^ab^ 35.6 ± 0.4 ^b^	0.54 ± 0.30 ^aA^ 0.29 ± 0.01 ^b^ 0.24 ± 0.02 ^c^	0.169 ± 0.01 ^aA^ 0.185 ± 0.01 ^b^ 0.200 ± 0.01 ^c^	3.23 ± 0.00 ^aC^ 3.23 ± 0.01 ^a^ 3.23 ± 0.01 ^a^	15.10 ± 0.10 ^aD^ 15.65 ± 0.07 ^b^ 16.73 ± 0.15 ^c^	1.01 ± 0.01 ^aD^ 1.05 ± 0.01 ^b^ 1.12 ± 0.02 ^c^

a, b, and c—samples marked with the same letters do not significantly differ statistically (in columns, between storage times) (*p* > 0.05); and A, B, C, and D—samples marked with the same letters (in columns, between SG addition of 0%, 10%, 20%, 30%, and 40%, without storage) do not differ statistically significantly (*p* > 0.05).

## Data Availability

The original contributions presented in this study are included in the article. Further inquiries can be directed to the corresponding author.
